# Bridging the Gap in Rhinoplasty Training: The Effectiveness of 3D Printed Models in Surgical Education

**DOI:** 10.1093/asj/sjaf045

**Published:** 2025-03-25

**Authors:** Umar Rehman, Natasha Polglase, David Kahn, Teoman Dogan, Santdeep Paun, Alwyn D’Souza, Rajan Uppal, Nicholas Eynon-Lewis, Matt Lechner

## Abstract

**Background:**

Rhinoplasty is a common facial plastic surgery procedure for both functional and aesthetic indications. The use of 3-dimensional (3D) models has been reported as a potential method for providing hands-on training for learning rhinoplasty without jeopardizing patient care.

**Objectives:**

The objective of this study was to develop and validate a novel model for rhinoplasty training.

**Methods:**

The rhinoplasty models were designed and produced with proprietary 3D printing technology. Face and content validity were assessed during a rhinoplasty course involving 53 surgeons. Criterion validity was evaluated in a training session with 20 surgical residents, measuring improvements in surgical skills after practice with the 3D models, with an objective structured assessment of technical skills (OSAT).

**Results:**

All surgeons (*n* = 53, 100%) stated that the rhinoplasty model aided in their learning and development. In all, 91% (*n* = 48) of surgeons rated the realism of the model as excellent or very good in comparison to cadaveric specimens. Assessment of criterion validity showed a statistically significant improvement in OSAT scores among surgical residents, increasing from a baseline of 11.7/40 (SD ± 1.80) to 21.6/40 (SD ± 1.79) post session (*P* < .0001).

**Conclusions:**

The 3D rhinoplasty models showed good content, face, and criterion validity, objectively improving residents’ surgical performance. Rhinoplasty 3D models may serve as a precadaveric training adjunct, equipping trainees with fundamental skills before cadaveric dissection, or as a primary training modality in countries with limited cadaver access. Therefore the models offer an innovative approach to training the next generation of rhinoplasty surgeons.

Surgical training allows surgeons to practice and hone surgical skills to achieve the best possible outcome for the patient.^1,2,3^ The traditional “see one, do one, teach one” approach to surgical training is no longer seen to be sufficient or acceptable for patient safety.^2,3^ Surgical training and education are evolving as technology has advanced.^2,4,5^ Simulation plays a vital role in expediting training and ensuring surgeons are safe practitioners.^2,4,5^

Rhinoplasty and septorhinoplasty are some of the most performed facial plastic surgery (FPS) procedures for both functional and aesthetic purposes.^6,7,8^ Rhinoplasty is considered one of the most technically demanding of all FPS procedures, and it carries a steep learning curve and high stakes for the patient, with the margin for error being narrow.^6,7,8^ The complexity often means that training opportunities available are limited, and gaining experience can prove to be difficult.^9,10,11^ Knowledge of rhinoplasty typically is attained from a variety of sources, including book chapters, didactic sessions, and video references. However, the mastery of technical skills is achieved through hands-on exposure, and obtaining sufficient exposure remains a challenge. Surgical residents or fellows are often not exposed to sufficient cases or given adequate time during their training to acquire skills for such procedures. Possible additional methods for surgeons to develop skills in rhinoplasty include cadaveric training, video-assisted training, observation in the operating room, and simulation.^7^

Anatomically accurate models act as an excellent tool for both the new and experienced surgeon, particularly in procedures in which residents have limited exposure during their training.^1,7,8^ Three-dimensional (3D) models have been reported as a potential method for hands-on training and learning rhinoplasty without jeopardizing patient care.^7,8^ Previous work has suggested that surgeons feel simulator models will help boost their confidence in rhinoplasties.^7,8^ Three-dimensional printing and modeling has a lower overall cost and avoids the ethical issues associated with cadaveric training.^5^

## METHODS

The study took place between November 2023 and February 2025. Model development, initial surgeon feedback, and expert interviews occurred between November 2023 and November 2024, followed by a pilot session assessing surgical skills in February 2025. The same model design was employed throughout the study.

### Three-Dimensional Printed Simulation Model Design

The rhinoplasty 3D simulation models were designed and produced by Fusetec (Adelaide, South Australia, Australia). These models were manufactured based on 3D printer technology from axial, coronal, and sagittal computed tomography (CT) scans of sinus surgery patients, with additional digital clay sculpting. A Stratasys Digital Anatomical Printer (Stratasys, Ltd, Rehovot, Israel) was utilized with digital anatomical creator materials that were a compound of multiple polymers, and Fusetec created its own digital materials with the software. For image segmentation Mimics (Materialise NV, Leuven, Belgium) was utilized, which is an industry standard software for segmentation. Netfabb (Autodesk, Inc., San Francisco, CA) was utilized for design and repair of files for advanced manufacturing. Utilizing materials from 3D anatomical printers, the models’ materials were developed to align with similar anatomical properties of nasal cartilages, such as elasticity and malleability.

**Figure 1.**
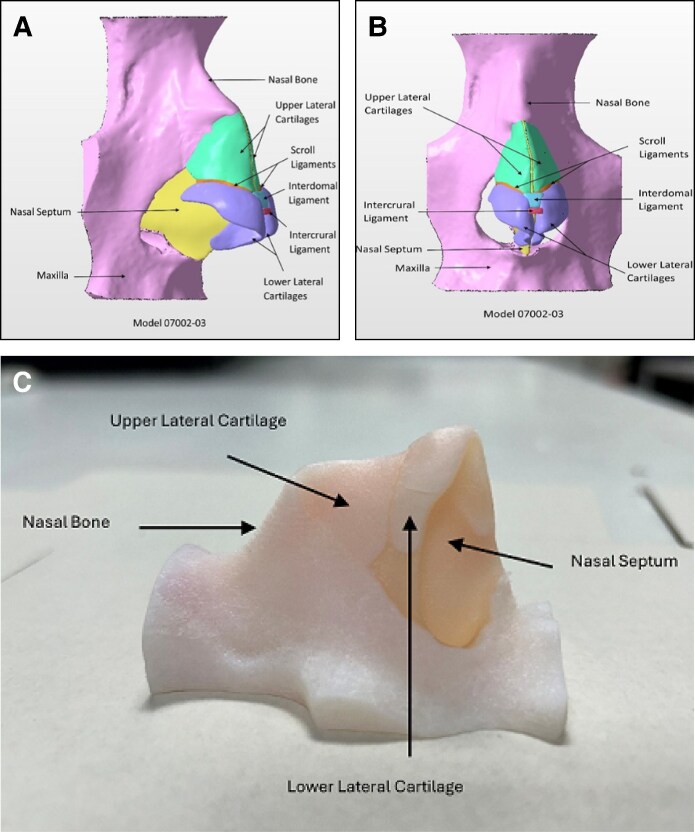
Anatomy of 3D model. (A) Lateral view. (B) Anterior view. The images illustrate a labeled stereolithography assembly of the rhinoplasty model from computed tomography (CT) scans of sinus surgery patients, with additional digital clay sculpting. (C) Lateral view of 3D printed model with anatomy highlighted. 3D, 3-dimensional.

Three different iterations of the rhinoplasty model (model 07002-01, model 07002-02, and model 07002-03; Figure 1) were developed, with all models comprised of cancellous and nasal bone, a septum, lower alar cartilages, upper alar cartilages, interdomal and intercrural ligaments, and scroll ligaments. Prominent features found across the 3 models were a dorsal hump and deviated septum. In addition, a rectangular 1 × 15 × 30 mm piece of lower alar cartilage was crafted to allow practicing surgeons to undertake a septal graft extension. The lower alar cartilage pathologies differed across the 3 models, resembling variations found in various rhinoplasty patients. All models were printed and prepared in Adelaide before being shipped to University College London, London, UK.

### Rhinoplasty Training Course

Face and content validity of the 3D models was evaluated in a surgical rhinoplasty course. Overall, 53 surgeons attended an in-person course focusing on techniques in rhinoplasty. This was a 1-day course that consisted of small group lectures, practical cadaveric dissection, observed procedures and techniques, and practical use of 3D printed rhinoplasty models. Faculty consisted of plastic surgery and otorhinolaryngology consultants and attending physicians experienced in rhinoplasty. The faculty-to-delegate ratio was 1:3 to aid in teaching and procedural steps. The 3D model simulation was delivered alongside practical cadaveric dissection, giving surgeons the chance to practice techniques and review anatomy before the cadaveric dissection. It also allowed faculty to explain basic principles and techniques before performing cadaveric dissection.

**Figure 2.**
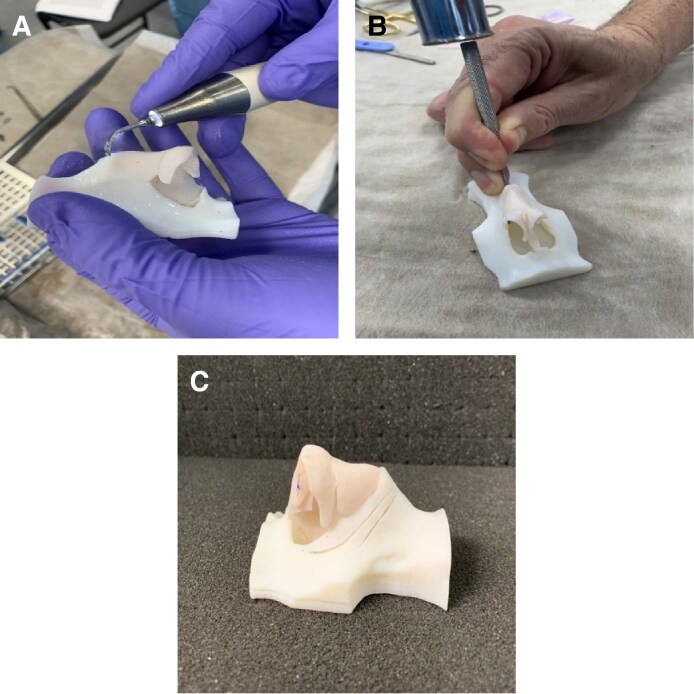
(A) Dorsal hump correction with Piezo technique. (B) Conventional manual technique for correction of dorsal hump. (C) Outline of lateral osteotomy performed by Piezo technique (columellar strut suture also depicted).

**Figure 3.**
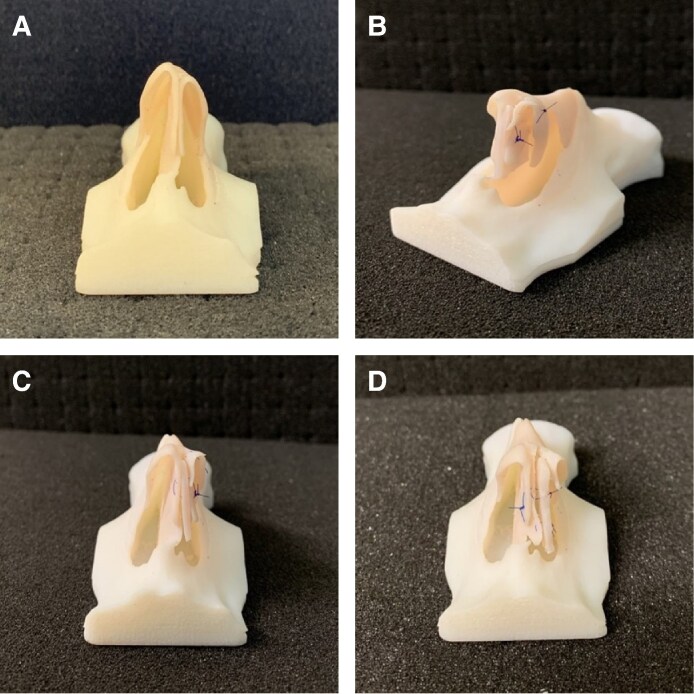
Correction of asymmetric nasal tip with 3D rhinoplasty model. (A) Asymmetric nasal tip (basal view). (B) Lateral crural steal and columellar strut (45-degree view). (C) Columellar strut and lateral crural steal (basal view). (D) Septal extension graft placement (basal view). 3D, 3-dimensional.

**Figure 4.**
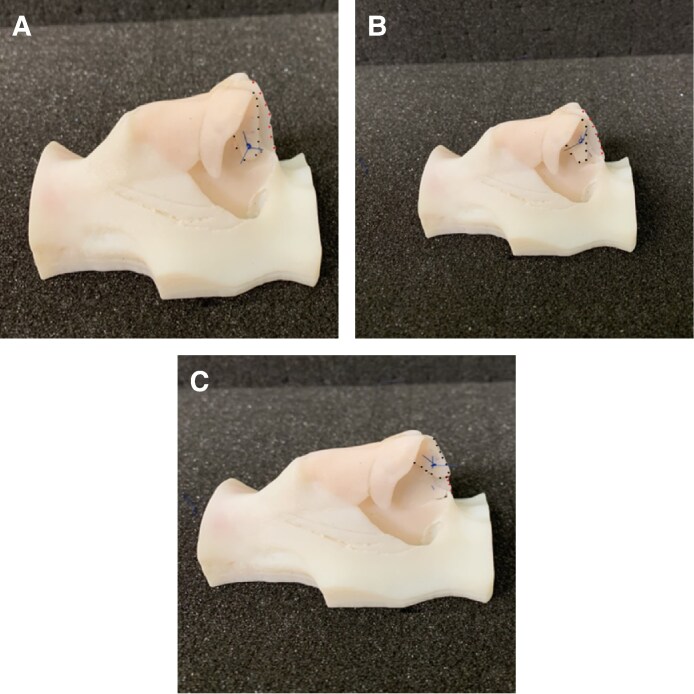
Tongue-in-groove technique with 3D model (right profile views). (A) New position of the medial crura with septal extension graft. (B) Exaggeration of medial crura position. (C) Final position of medial crura and excess septal extension graft trimmed (red dotted line indicates septal extension graft; black dotted line indicates medial crura). 3D, 3-dimensional.

Following completion of the course, surgeons were asked to complete anonymous paper surveys that adopted a combination of yes/no items and Likert scales to evaluate their experience with the 3D models in conjunction with cadaveric dissection to assess face validity (Appendix A, located online at https://doi.org/10.1093/asj/sjaf045). The 3D printed models allowed surgeons to perform several different rhinoplasty procedural steps, including dorsal hump reduction (Figure 2A, B), osteotomy (Figure 2C), nasal tip correction with crural steal (Figure 3A), columellar strut (Figure 3A, B), septal extension graft (Figure 3C) and tongue-in-groove techniques for nasal tip projection (Figure 4). Surgeons included in the study were residents and attending physicians in specialties including plastic surgery, otorhinolaryngology, and oral and maxillofacial surgery, seeking to gain further skills in rhinoplasty. Surgeons of all skill levels, from residents and trainees to attending physicians and consultants, were included. Content validity was assessed by having delegates rate the utility of the models for learning and training in rhinoplasty skills. No specific inclusion or exclusion criteria were employed, and all delegates attending the course who provided informed consent for their results to be a part of the research were included in the study.

### Expert Interview

Face validity was further evaluated by conducting semistructured interviews with experienced consultants and attending surgeons who regularly performed rhinoplasties as part of their clinical practice (Appendix B, located online at https://doi.org/10.1093/asj/sjaf045). This was to obtain further feedback on the realism and haptics as compared to standard clinical practice. Six consultant surgeons experienced in rhinoplasty were interviewed on their experience with the 3D models. Each surgeon utilized the model to perform the various techniques discussed earlier. No identifiable data were collected from the experts.

### Pilot Session Assessing Surgical Performance and Skill Development

Criterion validity was assessed by recruiting 20 surgical residents (postgraduate years 2 to 4) training in otorhinolaryngology (*n* = 13) or plastic surgery (*n* = 7) in the United Kingdom for a 3-hour pilot training session. This session was conducted independently of the rhinoplasty training course.

The session was delivered by attending rhinoplasty surgeons and included practical demonstrations followed by hands-on practice with 3D models. Surgical residents’ skills were evaluated with the objective structured assessment of technical skills (OSAT) tool, which has been employed and validated in previous research.^9,10^

The OSAT consists of a 7-item form with a 5-point rating scale linked to clear descriptors.^9,10^ Two independent attending surgeons completed the OSAT for each participant, and a mean score was calculated. Scores were recorded at the start and end of the session. The OSAT was employed because there is no rhinoplasty-specific assessment tool available appropriate for surgical residents. The purpose of the OSAT was to assess whether surgical skills transferable to rhinoplasty training had improved, including respect for tissue, time and motion efficiency, instrument handling, knowledge of instruments, procedural flow, and overall procedural knowledge.

Self-reported confidence levels were assessed before and after the course for each participant in 3 domains: understanding of nasal anatomy, understanding of septal extension grafts, and nasal tip suturing. Each domain was rated on a scale from 0 (no confidence) to 5 (very high confidence), with a maximum total score of 15 across all 3 items.

### Inclusion Criteria for Pilot Session

All participants were required not to have previously attended any other rhinoplasty or facial plastic surgery dissection courses. Additionally, all residents were required to have no more than 6 months of experience in rhinology or facial plastic surgery and be able to provide informed consent for their results to be part of the research.

### Statistical Analysis

The collected data were transferred and analyzed with GraphPad Prism 7 (GraphPad Software, San Diego, CA). Data were expressed as mean and standard deviation. An Anderson-Darling test was conducted to assess the normal distribution of the data. Data that followed a normal distribution (pre-course and post-course OSAT and confidence scores) were analyzed with parametric testing (paired *t* tests). Statistical significance was set at *P* < .05.

### Ethical Statement

Surgeons taking part in the survey or training session provided informed consent to participate in the study and have their data included as part of research by voluntarily completing the survey and attending the course. All data collected were anonymous, and no patient data were collected or employed as part of the qualitative research. In accordance with the ethical guidelines prevailing in the United Kingdom, an extensive ethical assessment was conducted with the UKRI Research Ethics Tool before initiation of our study. Ethical approval was not required, per the UKRI Research Ethics Tool.

### Aims

The purpose of this study was to develop and validate a 3D printed model for demonstrating multiple rhinoplasty techniques. Additionally, in the study we aimed to assess whether surgical performance improved following training with the novel 3D rhinoplasty models.

## RESULTS

### Breakdown of Participants

In total, 53 surgeons were surveyed to assess the face and content validity of the 3D printed models for rhinoplasty training. Fifteen surgeons were consultants or attending physicians, 35 were surgical residents or trainees, and 3 surgeons were postresidency fellows. The majority of surgeons (*n* = 44, 83.0%) were working or training in the United Kingdom, followed by Croatia (*n* = 2, 3.8%), the Netherlands (*n* = 1, 1.9%), Turkey (*n* = 1, 1.9%), USA (*n* = 1, 1.9%), Mexico (*n* = 1, 1.9%), Italy (*n* = 1, 1.9%), Ireland (*n* = 1, 1.9%), and Costa Rica (*n* = 1, 1.9%).

### Use of Cadaveric Dissection in Rhinoplasty Training

First, surgeons were asked about their experience with the cadaveric dissection. They were posed with the following yes or no question: Do you think that operating on cadavers made a significant contribution to the course? All surgeons (*n* = 53, 100%) responded yes. Next, they were asked whether operating on cadavers should be maintained in future courses. Again, all surgeons (*n* = 53, 100%) answered yes.

### Experience With 3D Printed Rhinoplasty Models

Surgeons were then able to practice surgical skills on the 3D printed models, and their feedback was then obtained. They were asked whether they were better able to apply surgical techniques and anatomy following practice with 3D printed models. Learning objectives covered a variety of areas, including anatomy of the nasal cartilages and ligaments, suturing technique on the nasal tip, applying septal extension grafts, understanding the tongue-in-groove technique, applying previous knowledge to current techniques, and performing suturing techniques to alter the shape of the nasal cartilages (Table 1).

**Table 1.** Surgeons on How 3D Models Help Achieve Course Learning Objectives

Prompt:	Answers (*n*, %)			
After having worked with the models do you feel the following learning objectives were met? *I am now better able to:*	Yes	Partially	No	Not performed
Understand the anatomy of the nasal cartilages and nasal ligaments.	48 (90.6%)	5 (9.4%)	0	0
Understand how each suturing technique affects the 3D configuration of the nasal tip.	42 (79.2%)	11 (20.8%)	0	0
Understand a septal extension graft more effectively and satisfactorily.	51 (96.2%)	2 (3.8%)	0	0
Understand the tongue-in-groove technique more effectively and satisfactorily.	34 (64.2%)	10 (18.8%)	7 (13.2%)	2 (3.8%)
Apply previous or new knowledge gained to current techniques and practices.	50 (94.3%)	3 (5.7%)	0	0
Perform rhinoplasty suturing techniques to alter the shape of the nasal cartilages.	46 (86.8%)	7 (13.2%)	0	0

3D, 3-dimensional.

All surgeons (*n* = 53, 100%) stated that the rhinoplasty model aided in their learning and development. Surgeons were asked to rate the realism of the model and the quality of the model for suturing in comparison to the cadaveric specimens. This was done with a Likert scale (1-5, from poor to excellent). With regard to the realism of the rhinoplasty model, 24 (45.3%) surgeons rated the model as excellent (5); 24 (45.3%) rated it very good (4); and 5 (9.4%) rated it good (3). When assessing surgeon experience with the quality of the rhinoplasty model for suturing, 24 (45.3%) surgeons rated this as excellent; 21 (39.6%) surgeons rated this as very good; 7 (13.2%) surgeons rated this as good; and 1 (1.9%) surgeon did not perform this activity.

Surgeons were asked whether they would prefer the rhinoplasty models over cadavers in future training. Overall, 29 (54.7%) surgeons stated that utilizing both cadavers and 3D models would be their preferred learning method for future courses; 19 (35.8%) surgeons preferred 3D models in future courses over cadavers; and 5 (9.4%) surgeons preferred to have only cadavers in future courses. When asked to justify their preference for 3D models over cadavers, surgeons’ responses were divided into themes that included reduced course costs (*n* = 15); increased course availability (*n* = 13); and individual models potentially available for each participant (*n* = 25).

### Expert Interview

Face validity was further assessed through feedback from 6 experienced rhinoplasty surgeons through semistructured interviews. In all, 83.3% (*n* = 5) had >15 years of experience performing rhinoplasties at an attending physician or consultant level, and 16.7% (*n* = 1) had 1 to 5 years of experience, with 83.3% (*n* = 5) performing 1 to 5 surgeries per month and 16.7% (*n* = 1) performing 11 to 15 surgeries per month as part of their clinical practice. Surgeons were then asked to rate the realism of the 3D model nasal cartilage and bone texture and tissue handling and haptic feedback with real tissue when performing cartilage suturing or nasal osteotomies; the results are depicted in Table 2.

**Table 2.** Rhinoplasty Surgeons Rate 3D Model Realism

Prompt:	Answers					
On a scale of 0-5 please rate the following items:	0 (Not realistic at all)	1	2	3	4	5 (Very realistic)
“Does the tissue texture of the nasal cartilage on the 3D printed model resemble that of real patients or cadavers during the suturing process?”	0	0	0	2 (33.3%)	2 (33.3%)	2 (33.3%)
“Does the pliability/flexibility of the nasal cartilage on the 3D printed model resemble that of real patients or cadavers when performing cartilage suturing?”	0	0	0	0	6 (100.0%)	0
“Does the haptic/tactile feedback from the nasal osteotomies performed on the models resemble that of real patients or cadavers?”	0	0	0	0	1 (16.7%)	5 (83.3%)
“Does the haptic/tactile feedback from the nasal cartilage suturing performed on the models resemble that of real patients or cadavers?”	0	0	0	1 (16.7%)	1 (16.7%)	4 (66.7%)

3D, 3-dimensional.

General feedback was very positive, with comments including “the bone is very realistic in particular”; “the bone is very similar to that in real patients”; “for a beginner surgeon I would recommend this model first, and then a cadaver”; and “this is an easy way to train juniors.” It was felt that the models could be improved by adding a soft tissue-mucosa overlay.

### Pilot Session Assessing Surgical Performance and Skill Development

Criterion validity was assessed by faculty rating residents’ surgical performance with the OSAT score at the start of the rhinoplasty training session and then again at the end of the 3- hour session. All residents (*n* = 20) demonstrated an improvement in the OSAT score following the session. The mean pre-course OSAT score was 11.7/40 (SD +/- 1.80) and the post-course OSAT score was 21.6/40 (SD +/-1.79) (*P* < .0001) (Figure 4).

Self-reported confidence also increased in all surgical residents, with the mean pre-course confidence reported to be 4.4/15 (SD +/-2.7) and the post-course confidence reported to be 9.35/15 (SD+/-2.1), *P* < .001 (Figure 5).

## DISCUSSION

Rhinoplasty is a complex and challenging procedure, with surgeons and those in training often having difficulty gaining exposure to the procedure.^7,8,11,12,13^ Surgeons worldwide, at various training stages, gave positive feedback on the 3D printed rhinoplasty models, finding them realistic compared to cadaveric specimens. The models were reported to aid in learning and skill development, demonstrating strong face and content validity. Additionally, incorporating the models into teaching sessions led to improved surgical skills, highlighting good criterion validity.

### Importance of Training in Rhinoplasty

The high demand for facial plastic surgery and particularly rhinoplasty highlights the motivation and need to assess and improve current training in rhinoplasty.^7,8,13,14^ Previous research demonstrates that rhinoplasty has the lowest confidence levels among residents in comparison to other aesthetic surgical procedures, such as breast surgery or abdominoplasty.^11^ In the literature, a large majority of residents have received training and learning in rhinoplasty by observing senior surgeons (reported in up to 92.5% of training methods) or through textbook independent learning (77.5%). The use of simulation models is less common.^7,8^

Anastakis et al described cadaver training as “the gold standard for technical skills training.”^15^ It allows surgeons to train with mistakes being performed in a learning environment without patient safety consequences. In our study all surgeons felt that cadavers made a significant contribution to learning in the course and should be maintained in the future. They clearly agreed with the utility of cadaveric dissection in surgical training, consistent with previous literature.^15,16^

Jacovella et al demonstrated significant higher scores related to knowledge, attitude, and skills in rhinoplasty in residents undergoing cadaveric training compared to those that did not.^16^ However, cadaveric dissection, although proven to show benefit for surgical training in rhinoplasty, is not without its limitations. Limitations with cadavers include the costs, maintenance, lack of cadaveric donations (particularly in the UK), difficulty with continuing to practice techniques beyond the course, and lack of widespread availability of courses.^17^ Zammit et al reported that only 20.3% of residents gained exposure to rhinoplasty through cadaveric dissection, likely attributed to the financial costs involved in the training and lack of widespread availability and limited places in cadaveric courses.^7^

### Simulation Models in Rhinoplasty

In this study simulation models provided a realistic model for training surgeons in rhinoplasty techniques. The models in this study were reported by most surgeons to be realistic compared to cadaveric anatomy, with 90.6% of surgeons rating the realism as excellent or very good. Expert rhinoplasty surgeons described the models to be realistic in flexibility, texture, and tactile feedback. The models in this study allowed surgeons to better understand the anatomy of the nasal cartilages and ligaments, and all surgeons felt that the models aided in their learning and development. Most surgeons (90.6%) felt that future courses should incorporate a combination of 3D printed models together with cadaveric dissection or 3D models alone. Of those who preferred the 3D models alone for future courses, this was attributed to the anticipated reduced course fees, wider availability of courses, and having individual models for each delegate.

In this study, surgeons were able to practice a range of rhinoplasty steps with the models. With the anatomical differences between the 3 models, surgeons were allowed to appreciate the complexity of the surgery and the variation of techniques required based upon an individual patient. Key aspects of the rhinoplasty procedure could be broken down and repeated. A clear majority of surgeons were better able to understand septal extension grafts (96.2%), how the suturing techniques altered the shape of the nasal cartilages (86.8%) and nasal tip configuration (79.2%), and the tongue-in-groove technique (64.2%).

As 3D models become more accessible, surgical trainees can practice at home or work with senior surgeons providing real-time feedback. These models allow for on-demand pathology creation, enabling training without the risks of pathogens or the need for protective equipment in non-wet lab conditions.

### Nasal Tip Suture Placement Simulation

Illustrations of lower and lateral cartilages often fail to show that the medial and lateral crura are in different planes, making them not truly 2-dimensional.^18^ Minor suture repositioning in the lower lateral cartilages can yield significantly different results. Success in nasal tip surgery depends on a 3D understanding of the lower lateral cartilage and suture placement. This can be challenging for less experienced rhinoplasty surgeons.^18,19^ This was addressed with the models, with 90.1% of surgeons gaining a better understanding of the impact of suture placement on the nasal tip's 3D configuration, allowing delegates to grasp the intricacies of nasal tip suturing.

### Future Directions

Future work will aim to develop the models further, incorporating soft tissue elements and encompassing the entirety of the anatomy. Further work to validate the models will require comparing performance scores before and after training with 3D models alone, with a control group undergoing only cadaveric training. Moreover, the 3D models may serve as an adjunct to cadaveric training, acting as a pre-cadaveric tool to help trainees develop fundamental skills and concepts before cadaveric dissection, helping with skill acquisition. This approach may maximize the learning opportunities during cadaveric dissection. Additionally, countries with limited access to cadavers can employ 3D models for training, which may offer a more sustainable, practical, and cost-effective alternative to attending international dissection courses. Finally, longitudinal retention of rhinoplasty skills can be enhanced with 3D models, with dissection courses supplemented by these models to support skill retention beyond the cadaveric course.

### Limitations

This study had several limitations, including that each respondent involved in the face and content validation had varied surgical experience and was at a different stage of their surgical career. The nature of their experience and their familiarity with rhinoplasty were not assessed in this study. The sample size utilized in the present study may appear limited but is larger than in previous literature related to 3D models in rhinoplasty and aesthetic surgery education.^20,21,22^

## CONCLUSIONS

The novel 3D rhinoplasty models demonstrated strong face, content, and criterion validity. All surgeons agreed that the models enhanced learning and skill development, helping them better achieve the course's learning objectives. Additionally, utilization of the models led to a significant improvement in surgical residents’ operative skills. Future courses should consider integrating 3D simulation models alongside, or even before, traditional cadaveric dissection. This would provide delegates with more hands-on experience in rhinoplasty and better prepare them for cadaveric dissection, allowing them to maximize the learning opportunity. Furthermore, the 3D models can serve as a primary training modality in countries with limited cadaver access, ensuring accessible training. The proprietary 3D rhinoplasty model offers an innovative approach to training the next generation of rhinoplasty surgeons.

## Supplemental Material

This article contains supplemental material located online at https://doi.org/10.1093/asj/sjaf045.

## Supplementary Material

sjaf045_Supplementary_Data
