# Supplementary material for: Bridging the Gap in Rhinoplasty Training: The Effectiveness of 3D Printed Models in Surgical Education
Source: Aesthet Surg J. 2025 Mar 25;45(7):655–61. doi: 10.1093/asj/sjaf045 (PMC12168444; doi:10.1093/asj/sjaf045)
Supplement: sjaf045_Supplementary_Data [file sjaf045_supplementary_data.docx]

**Appendix A. Questionnaire for Delegates to Assess Quality of 3D Models.**

1. **What stage of training are you?**

Intern/Foundation doctor 🔾 Core Surgical Trainee/Junior Resident 🔾 Intermediate Resident ST3-5 🔾 Senior Resident ST6-8 🔾 Attending 4 🔾 Fellow 🔾 Other 🔾

If Other, please state: _____________________________________________________________________________________

1. **Which country are you currently working in?** ________________
2. **Prior to the course how many functional or cosmetic septorhinoplasties have you performed as primary surgeon ? (Please circle)**

0 1-2 3-4 5-6 6-7 >8

1. **Do you think that operating on cadavers made a significant contribution to the course?**

Yes 🔾 No 🔾 Unsure 🔾

1. **Do you think operating on cadavers should be continued on future courses?**

Yes 🔾 No 🔾 Unsure 🔾

1. **On a score of 1 – 5 with 1 = poor and 5 = excellent, how would you rate the overall realism of the Rhinoplasty Model compared to cadavers and real patients?**

Poor 🔾 Average 🔾 Good 🔾 Very good 🔾 Excellent 🔾

1. **On a score of 1 – 5 with 1 = poor and 5 = excellent,** **How would you rate the overall quality of suturing on the Rhinoplasty Model compared to cadavers and real patients?**

Poor 🔾 Average 🔾 Good 🔾 Very good 🔾 Excellent 🔾 Not performed 🔾

1. **Please state if the use of 3D models contributed to achieving the following learning objectives.**

| **After having worked with the models do you feel the following learning objectives below were met**  ***I am now better able to:*** | **Yes (n, %)** | **Partially** | **No** | **Not performed** |
| --- | --- | --- | --- | --- |
| Understand the anatomy of the nasal cartilages and nasal ligaments |  |  |  |  |
| Understand how each suturing technique affects the 3D configuration of the nasal tip |  |  |  |  |
| Understand a septal extension graft more effectively and satisfactorily |  |  |  |  |
| Understand the tongue in groove technique more effectively and satisfactorily |  |  |  |  |
| Apply prior or new knowledge gained into current techniques and practices |  |  |  |  |
| Perform rhinoplasty suturing techniques to alter the shape of the nasal cartilages |  |  |  |  |

1. **Did the 3D rhinoplasty models aid in your learning and development of rhinoplasty skills?**

Yes 🔾 No 🔾 Unsure 🔾

1. **Are there any characteristics which could be altered to improve the Rhinoplasty Model?**

__________________________________________________________________________________________________________

__________________________________________________________________________________________________________

__________________________________________________________________________________________________________

1. **Would is your preferred method of learning Rhinoplasty skills for future courses?**

Cadavers only 🔾 3D models only 🔾 Cadavers and 3D models 🔾 Other (Please specify)____________ 🔾

1. **Would you encourage your local department to obtain 3D models for rhinoplasty training**

Yes 🔾 No 🔾 Maybe 🔾

1. **If you prefer using 3D models over cadavers, please provide your reason below.**

____________________________________________

**Appendix B. Semi-structured Interview Questions for Expert Surgeons.**

1. **How many years of experience do you have performing rhinoplasties as a consultant/attending surgeon ?**
2. <1 year b. 1-5 years c. 6-10 years d. 11-15 years e. >15 years
3. **How many rhinoplasty procedures (functional and/or cosmetic) do you perform on average each month?**

Nil b. 1-5 c. 6-10 d. 11-15 e. >15

1. **Using the scale below, from 0 (Not realistic at all) to 5 (Very realistic), please rate the 3D models in each aspect compared to real patients or cadavers.**

| **On a scale of 0-5 please rate the following items** | **0 (Not Realistic at all)** | **1** | **2** | **3** | **4** | **5 (Very Realistic)** |
| --- | --- | --- | --- | --- | --- | --- |
| “Does the tissue texture of the nasal cartilage on the 3D printed model resemble that of real patients or cadavers during the suturing process” |  |  |  |  |  |  |
| “Does the pliability/flexibility of the nasal cartilage on the 3D printed model compared to real patients or cadavers when performing cartilage suturing.” |  |  |  |  |  |  |
| “Does the haptic/tactile feedback from the nasal osteotomies performed on the models as compared to real patients/cadavers” |  |  |  |  |  |  |
| “ Does the haptic/tactile feedback from the nasal cartilage suturing performed on the models as compared to real patients/cadavers. |  |  |  |  |  |  |

1. **Do you have any general feedback regarding the 3D rhinoplasty models?**
